# An economic evaluation of universal and targeted case-finding strategies for identifying antenatal depression: a model-based analysis comparing common case-finding instruments

**DOI:** 10.1007/s00737-023-01377-2

**Published:** 2023-10-18

**Authors:** Elizabeth M. Camacho, Gemma E. Shields, Emily Eisner, Elizabeth Littlewood, Kylie Watson, Carolyn A. Chew-Graham, Dean McMillan, Shehzad Ali, Simon Gilbody

**Affiliations:** 1 School of Health Sciences, University of Manchester, Manchester, UK; 2 Institute of Population Health, University of Liverpool, Liverpool, UK; 3 Manchester Mental Health NHS Foundation Trust, Manchester, UK; 4 Department of Health Sciences, University of York, York, UK; 5 Manchester University NHS Foundation Trust, Manchester, UK; 6 School of Medicine, Keele University, Keele, UK; 7 Hull York Medical School and Department of Health Sciences, University of York, York, UK; 8 Schulich School of Medicine & Dentistry, Western University, London, Canada

**Keywords:** Antenatal depression, Case finding, Cost-effectiveness

## Abstract

Half of women with depression in the perinatal period are not identified in routine care, and missed cases reflect inequalities in other areas of maternity care. Case finding (screening) for depression in pregnant women may be a cost-effective strategy to improve identification, and targeted case finding directs finite resources towards the greatest need. We compared the cost-effectiveness of three case-finding strategies: no case finding, universal (all pregnant women), and targeted (only pregnant women with risk factors for antenatal depression, i.e. history of anxiety/depression, age < 20 years, and adverse life events). A decision tree model was developed to represent case finding (at around 20 weeks gestation) and subsequent treatment for antenatal depression (up to 40 weeks gestation). Costs include case finding and treatment. Health benefits are measured as quality-adjusted life years (QALYs). The sensitivity and specificity of case-finding instruments and prevalence and severity of antenatal depression were estimated from a cohort study of pregnant women. Other model parameters were derived from published literature and expert consultation. The most cost-effective case-finding strategy was a two-stage strategy comprising the Whooley questions followed by the PHQ-9. The mean costs were £52 (universal), £61 (no case finding), and £62 (targeted case finding). Both case-finding strategies improve health compared with no case finding. Universal case finding is cost-saving. Costs associated with targeted case finding are similar to no case finding, with greater health gains, although targeted case finding is not cost-effective compared with universal case finding. Universal case finding for antenatal depression is cost-saving compared to no case finding and more cost-effective than targeted case finding.

**Supplementary information:**

The online version contains supplementary material available at 10.1007/s00737-023-01377-2.

## Introduction

Antenatal depression is depression that occurs during pregnancy. The global prevalence is estimated to be between 5 and 65% (Underwood et al. 2016; Dadi et al. 2020; Yin et al. 2021). A literature review reported that 39% of women who experienced antenatal depression also had postnatal depression and 47% of women with postnatal depression had also had antenatal depression (Underwood et al. 2016). The evidence suggested that depression is more common during pregnancy than postnatally, and that postnatal depression is often a continuation of antenatal depression. Antenatal depression is also associated with an increased risk of low birth weight (1.40 times higher) and preterm birth (1.49 times higher) when compared with mothers who were not depressed (Dadi et al. 2020). There is consistent evidence that perinatal mental illness is not well-identified or treated in current systems. For example, a prospective cohort study of pregnant women in London, England, between 2014 and 2016 reported that only a third of women with a diagnosable mental disorder at the first antenatal appointment had any contact with mental health services during pregnancy or in the early postnatal period (Lee-Carbon et al. 2022).

One way of improving the identification of illness is to implement a screening programme. When the screening instrument used is not a formal diagnostic test, the term ‘case finding’ can be used. We subsequently refer to case finding for antenatal depression in this paper. A recent review of perinatal depression screening recommendations in Organisation for Economic Co-operation and Development (OECD) member countries reported that while there were some exceptions, most publications identified endorsed screening for perinatal depression (El-Den et al. 2022). Guidelines from the National Institute for Health and Care Excellence (NICE) in England for antenatal and postnatal mental health recommend that at all contacts during pregnancy and the early postnatal period, healthcare providers should ‘consider’ asking women two probing questions related to depression (known as the Whooley questions (Whooley et al. 1997)) (National Institute of Health and Care Excellence (NICE) 2014). Yet the guidelines stop short of recommending systematic case finding.

A growing body of evidence suggests some women experience direct and indirect discrimination in the context of maternal healthcare (e.g. migrant populations and women from minority ethnic groups) (Higginbottom et al. 2019; MacLellan et al. 2022). The non-specific recommendation to ‘consider’ asking about mental health is an additional opportunity for health inequalities to be perpetuated within the system. A sequential mixed-methods study in England highlighted inequalities in the identification of perinatal mental health difficulties (Darwin et al. 2022). A specific recommendation from the research was that perinatal mental health should be a part of core healthcare business. Case finding is an evidence-based example of how this could be operationalised in practice, such that mental health checks become part of routine antenatal care.

A key barrier to implementing a universal case-finding strategy may be scarcity of healthcare practitioners and limitations on the time they can spend with patients. To manage competing demands on their time, practitioners need to prioritise when, and to whom, they ask case-finding questions. This would involve making an implicit judgement on an individual’s risk of developing antenatal depression. Consideration of risk factors can be used to systematically target healthcare resources to where the need is greatest and/or where they are most likely to have a benefit. There are established risk factors for antenatal depression which include low level of education, low income, unplanned pregnancy,(personal or family) history of mental illness, and low social and/or partner support (Dadi et al. 2020; Yin et al. 2021; Míguez and Vázquez 2021). A targeted case-finding strategy (whereby only women who are identified as being at high-risk of antenatal depression would complete a case-finding instrument) would require fewer resources to implement than a universal strategy and so may be more appealing to decision-makers and healthcare professionals. However, it may mean that some women with depression who are characterised as low risk may be less likely to be identified.

The aim of this study is to assess the cost-effectiveness of targeted and universal case finding for antenatal depression. We compared targeted and universal case finding with each other and standard care (no case finding).

## Materials and methods

### Economic decision-analytic model

**Fig. 1:**
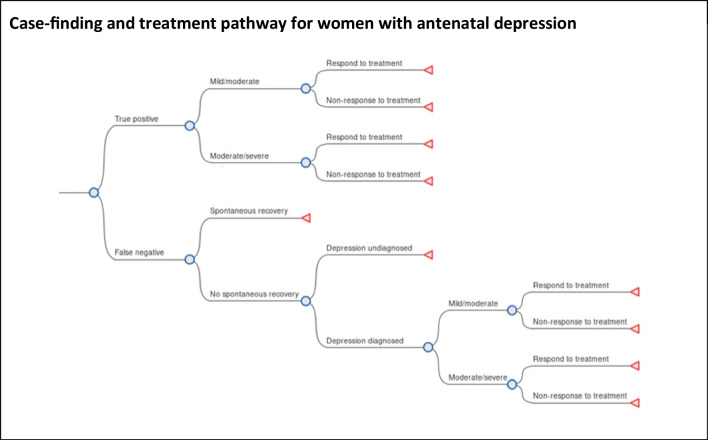
Case finding and treatment pathway for women with antenatal depression

The decision tree model used in a previously published cost-effectiveness analysis of universal antenatal depression case finding was adapted for the current analysis (National Institute of Health and Care Excellence (NICE) 2014, p. 192; Littlewood et al. 2018). There were three stages in the tree: risk-stratification (for the targeted approach only), case finding, and treatment. The risk-stratification stage simply directs those who are high-risk along the case-finding pathway and those who are low-risk towards standard care (i.e. no case finding) (Figure S1). The case-finding and treatment stages of the model are shown in Fig. 1 for women with antenatal depression. This pathway was the same for all strategies including the no case-finding option. The outcomes relating to identification for depressed women were true positive and false negative. The treatment stage included chance nodes for depression severity, treatment response, and spontaneous recovery and subsequent identification of depression in women with a false negative case-finding outcome. For women with a false negative case-finding outcome who do not spontaneously recover from antenatal depression, there is a chance that they are subsequently identified as depressed by their primary care physician/general practitioner (GP). There is only an identification stage for non-depressed women which composes of two branches: true negative and false positive. The branches for non-depressed women are shown in supplementary material (Figure S2) alongside those for depressed women from Fig. 1 as a complete picture.

The parameters for our model were predominantly derived by conducting secondary analysis of data from the BaBY PaNDA study. BaBY PaNDA was an observational longitudinal cohort study of pregnant women in Yorkshire, England, who were followed until one year postpartum (Littlewood et al. 2018). The full protocol for the BaBY PaNDA study has been published previously (Littlewood et al. 2016). Through secondary analysis of individual level data, we were able to derive specific model parameters that incorporated heterogeneity in risk factors for antenatal depression. Additional model parameters were derived from published literature. We identified source studies through targeted literature searching and citation searching of source papers used in the previous models developed by NICE and the BaBY PaNDA team (National Institute of Health and Care Excellence (NICE) 2014, p. 192; Littlewood et al. 2018). Expert opinion was also sought on all model parameters to ensure that they were reasonable and reflected current practice and lived experience. The experts consulted were members of the study team which included a GP, psychiatrist, clinical psychologist, and an expert by lived experience of perinatal depression. External to the study team, two additional GPs and three experts by lived experience of perinatal mental illness were consulted.

A full description and summary of the model parameters are reported in Table 1. The time horizon for the model was from the point of case finding at 20 weeks gestation (this was when data on case-finding instruments were collected for the BaBY PaNDA study) until 20 weeks later (i.e. approximating 40 weeks gestation at birth). The perspective for the cost-effectiveness analysis was the English NHS and social care services, in line with NICE guidance (NICE 2022). The price year was 2021, and the currency was British pounds (£). The model included costs for the administration and scoring of the case-finding instruments, additional assessment of cases (either by GPs or midwives), treatment (pharmacological, psychological, or both), and monitoring of women identified as having antenatal depression. For mild-to-moderate depression, costs included treatment with facilitated self-help and monitoring (£273). For moderate-to-severe depression, costs included treatment with either intensive psychological therapy (£910), the anti-depressant sertraline (£300), or both (£935), plus monitoring. The measure of health benefit was quality-adjusted life years (QALYs), derived from the EQ-5D-3L (The EuroQoL Group n.d.) (which was collected as part of the BaBY PaNDA study) and index values for the UK (Dolan 1997). As the time horizon for the model was less than 1 year, no discounting of costs or outcomes was required.

**Table 1:** Model parameters

Parameter	Value	Source
Probabilities		
Prevalence of risk factors		
Proportion of sample in high-risk sub-group	55.8% (95% CI 50.7 to 60.7%)	Derived from BaBY PaNDA data
Prevalence of depression		
Whole sample	10.3% (95% CI 7.4 to 13.7%)	Derived from BaBY PaNDA data
High risk sub-group	17.1% (95% CI 12.2 to 22.7%)	
Low-risk sub-group	1.7% (95% CI 0.4 to 5.0%)	
Severity of depression		
Moderate to severe	30% (SE = 0.030)	Derived from BaBY PaNDA data — proportion with CIS-R confirmed depression scoring moderately severe or above on PHQ-9
Mild to moderate	70% (SE = 0.070)	Derived from BaBY PaNDA data — inverse of above
Treatment received		
Mild/moderate depression	100% facilitated self help	NICE model (National Institute of Health and Care Excellence (NICE) 2014)
Moderate to severe depression	intensive psychological therapy only 12.8% (95% CI 9.2 to 17.1%) pharmacological therapy only (sertraline) 52.3% (95% CI 46.5 to 58.1%) both 34.9% (95% CI 29.5 to 40.6%)	Prady et al (2016) (Prady et al. 2016)
Probability of no recovery		
No spontaneous recovery of false negatives	0.65 (SE 0.065)	Brown et al (2021) Cochrane Review (placebo — response or remission) (Brown et al. 2021); also NICE model (TAU — diagnosis or symptomology) (National Institute of Health and Care Excellence (NICE) 2014)
Facilitated self-help	Probability of recovery (TAU) 0.38 RR of recovery compared to TAU 1.59 (95% CI 1.33 to 1.90) Probability of recovery (FSH) 0.60 (95% CI 0.51 to 0.72) Probability of no recovery 0.40 (95% CI 0.28 to 0.49)	Lin et al (2018) (Lin et al. 2018)
Intensive psychological therapy	Probability of no recovery (TAU) 0.64 RR of no recovery compared to TAU 0.69 (95% CI 0.56 to 0.85) Probability of no recovery 0.44 (95% CI 0.36 to 0.55)	NICE model (National Institute of Health and Care Excellence (NICE) 2014)
Pharmacological therapy	Probability of response (placebo) 0.43 RR of recovery for antidepressants versus placebo 1.27 (95% CI 0.97 to 1.66) Probability of recovery (treatment) 0.54 (95% CI 0.41 to 0.71) Probability of no recovery 0.46 (95% CI 0.29 to 0.59)	Brown et al 2021 Cochrane Review (Brown et al. 2021)
Combined IPT and pharmacological therapy	Probability of recovery (placebo) 0.43 RR of recovery for combined versus placebo 1.47 (95% CI 1.20–1.75) Probability of recovery 0.63 (95% CI 0.51 to 0.75) Probability of no recovery 0.37 (95% CI 0.25 to 0.49)	Brown et al 2021 Cochrane Review (Brown et al. 2021) Cuijpers et al 2020 (Cuijpers et al. 2020)
Identification of depression by GP		
Probability of visiting GP at least once — high risk	0.780 (95% CI 0.721 to 0.830)	Derived from BaBY PaNDA data
Probability of visiting GP at least once — whole sample	0.722 (95% CI 0.675 to 0.765)	
Probability of visiting GP at least once — low risk	0.642 (95% CI 0.565 to 0.712)	
Identification by GP — all FNs who do not spontaneously recover visit GP halfway through follow-up period	0.10 (sensitivity analysis 0.05 and 0.25)	Expert opinion
Sensitivity and specificity of standard care case identification		
Sensitivity	50.1% (95% CI 41.3 to 59.0%)	Mitchell et al (2009) (Mitchell et al. 2009)
Specificity	81.3% (95% CI 74.5 to 87.3%)	Mitchell et al (2009) (Mitchell et al. 2009)
Costs		
Cost of screening/case finding		
Whooley questions	0.71 min (SE = 0.045) + 1 min for scoring Midwife time — £55/h; £0.92/min (Band 6) Total cost = £1.57	BaBY PaNDA model (Littlewood et al. 2018) PSSRU (Jones et al. 2021)
EPDS or PHQ-9	1.54 min (SE = 0.055) + 2 min for scoring EPDS Midwife time — £55/h; £0.92/min Total cost = £3.26 PHQ-9 assumed to take same time as EPDS	BaBY PaNDA model (Littlewood et al. 2018) PSSRU (Jones et al. 2021)
Further assessment of positive cases (true and false) — no impact on treatment as a result of this assessment	1 h Midwife time — £55/h; £0.92/min Total cost = £55	NICE — cost of midwife time to conduct further assessment included in model. (National Institute of Health and Care Excellence (NICE) 2014)
Standard care case identification (true and false positives)/later identification of false negatives	9.22 min (one GP consultation) = £39.23 (SE = 3.9)	PSSRU (Jones et al. 2021) SE assumed
Treatments		
Facilitated self-help (for mild to moderate depression)	Seven sessions (25 min each = 175 min in total) provided by psychological wellbeing practitioner (band 5 — £41/working hour; assuming 4:1 ratio of face-to-face time and other activities = £51/h face-to-face contact; £0.85/min); plus guided self-help manual costing £7; plus 3 consultations with GP (£39.23 each) Total cost = £273.44	NICE model (National Institute of Health and Care Excellence (NICE) 2014) Mavranezouli et al. (2020) (Mavranezouli et al. 2020) PSSRU — scientific and professional staff (Jones et al. 2021)
Intensive psychological therapy (for moderate to severe depression)	Eight sessions (IQR 6 to 11 sessions) (55 min each = 440 min in total) provided by Clinical psychologist or Counsellor (specialist) (band 7 — £65/working hour; assuming 60:40 ratio of face-to-face time and other activities = £108/h face-to-face contact; £1.80/min); plus 3 consultations with GP (£39.23 each) Total cost = £909.69	Cuijpers et al. (2023) — number of sessions (Cuijpers et al. 2023) NICE model — session duration (National Institute of Health and Care Excellence (NICE) 2014) Mavranezouli et al. 2020) — unit costs (Mavranezouli et al. 2020) PSSRU — scientific and professional staff — unit costs (Jones et al. 2021)
Pharmacological therapy	Initial therapy with sertraline for 8 weeks plus 6 months maintenance therapy — 34 weeks. 28 × 50 mg tablets o.d. (£2.85/28 tablets; 9 packets) = £25.65Monitoring: 7 consultations with GP (£39.23 each) Total cost = £300.26	NICE model (National Institute of Health and Care Excellence (NICE) 2014) Expert opinion NHS Electronic Drug Tariff (2021 average cost) (NHS Prescription Services 2021) PSSRU (Jones et al. 2021)
Combined IPT and pharmacological therapy	Costs of IPT and GP follow-up (£909.69) plus sertraline prescription (£25.65). Total cost = £935.34	
False positive cases	20% of cost of FSH — assume treatment started by all would be FSH Total cost = £54.69	NICE model (National Institute of Health and Care Excellence (NICE) 2014)
Utility values		
Whole sample	Non-depressed: 0.910 (SE 0.01); *n* = 349 Decrement for mild depression: 12% Decrement for mod/severe depression: 26%	Derived from BaBY PaNDA data
High-risk sub-group	Non-depressed: 0.869 (SE 0.01); *n* = 188 Mild depression: 0.765 Mod/severe depression: 0.643	
Low-risk sub-group	Non-depressed: 0.910 (SE 0.01); *n* = 161 Mild depression: 0.801 Mod/severe depression: 0.673	
False positives	No utility decrement [2% reduction as sensitivity analysis]	-
Recovery post-treatment — true positives	Linear improvement over course of therapy Pharmacological (8 weeks) IPT (8 weeks) FSH (8 weeks)	As NICE and BaBY PaNDA models (National Institute of Health and Care Excellence (NICE) 2014, p. 192; Littlewood et al. 2018)
Spontaneous recovery — false negatives	Linear improvements over 7 weeks post screening	
No spontaneous recovery — false negatives	Continue as depressed, unless identified in routine care	
No response to treatment	Continue as depressed	
Depression not diagnosed	Continue as depressed	

*TAU* treatment as usual, *PSSRU* Personal Social Services Research Unit — unit costs of health and social care report

### Identifying high-risk women

A number of risk factors for PND have been consistently identified in published literature (Hutchens and Kearney 2020; Dadi et al. 2020; Míguez and Vázquez 2021). Three known risk factors that are routinely collected in antenatal care and can be assessed easily are age (< 20 years), history of anxiety, and history of depression. In the BaBY PaNDA study, participants self-reported information on these factors and so we used these to identify high-risk women in that cohort. We also explored a fourth risk factor, for which there is considerable evidence in the literature, that can be broadly described as ‘difficult life events’. This includes relationship breakdown, domestic violence, and unplanned pregnancies. As part of the BaBY PaNDA study, participants completed the List of Threatening Events/Experiences Questionnaire (LTE-Q) (Brugha et al. 1985) which we also used to define antenatal depression risk. In our model, anyone who had at least one life event or at least one of the other three risk factors was classified as being at high-risk for developing antenatal depression.

### Case-finding strategies

Seven case-finding strategies were considered: four one-stage strategies and three two-stage strategies. The one-stage strategies were the Edinburgh Postnatal Depression scale (EPDS) (Cox et al. 1987) (with thresholds of ≥ 10 and ≥ 13), the Whooley questions (a ‘yes’ response to either question indicates possible depression) (Whooley et al. 1997), and the PHQ-9 (with a threshold of 10) (Kroenke et al. 2001). The two-stage strategies all included the Whooley questions as the first stage, followed by either the EPDS (both thresholds) or the PHQ-9.

The sensitivity and specificity of each strategy was taken from the high-risk sub-group of the BaBY PaNDA cohort, which was assessed against a diagnostic gold standard clinical assessment of depression, the Clinical Interview Schedule — Revised (CIS-R) (Lewis et al. 1992). The main aim of the case-finding program is to identify more cases of antenatal depression. As such, strategies with a sensitivity of less than 70% were not considered to perform to an acceptable level (given that a sensitivity of 50% is no better than chance) and so were not included in the analysis. There are resources consumed when false positive cases are diagnosed and treated and so it is important to minimise this outcome. We have assumed that over-identification is preferable to under-identification of cases and so strategies with a specificity (i.e. ability to correctly detect non-cases) of less than 60% were considered not to perform to an acceptable level and so were excluded. The sensitivity and specificity of the strategies are reported in supplementary material (Table S1). The strategies included in our model were as follows: EPDS with a threshold of ≥ 10 (subsequently referred to as EPDS-10), the Whooley questions, and a two-stage strategy where a positive response to the Whooley questions was followed by the PHQ-9.

### Cost-effectiveness analysis

The costs, outcomes, and probabilities were entered into the model to estimate incremental cost-effectiveness ratios (ICERs) for a series of comparisons. The first stage was to estimate the relative cost-effectiveness of the three different case-finding strategies (EPDS-10, Whooley questions, and Whooley/PHQ-9) within the high-risk group of women. This was then repeated for the whole sample. The most cost-effective strategy was then used to compare the cost-effectiveness of case finding to no case finding among high-risk women only. Finally, three strategies were compared: no case finding, universal case finding, and targeted case finding (i.e. case finding with high-risk women only). In the ‘no case-finding’ comparator and for low-risk women in the targeted case-finding approach, women can only be identified as having antenatal depression if they visit a GP (primary care physician). Probabilistic analyses were conducted to quantify decision uncertainty in the analysis. The value for each of the probabilities and utilities in the primary (deterministic) model was randomly selected 10,000 times from a distribution around the values. This generated a 95% confidence interval around the mean cost and mean QALYs. Beta distributions were assumed for probabilities and utility values. Unit costs were assumed to be fixed (as in the BaBY PaNDA model (Littlewood et al. 2018)). The results from these simulations were used to calculate the probability that the different strategies would be cost-effective at willingness to pay thresholds of £0, £20,000, and £30,000/QALY. Decision-makers in England cite a cost-effectiveness threshold of £20,000/QALY, whereby interventions costing less than this amount to improve health by one QALY are considered to be cost-effective (NICE 2022).

To provide an estimate of the resources required to implement a nationwide PND case-finding programme, total costs and QALYs were calculated based on the approximate number of women who give birth per year in England and Wales (*n* = 600,000, 2020 data) (Office for National Statistics 2020).

The model was built by one health economist and was validated separately by two other health economists (one who was part of the study team and one external person). Validation included checks around face validity, logical consistency (including using extreme and null values, and tracing patients throughout the model), and cross-validation testing using the results of other studies.

### Sensitivity analyses

Sensitivity analyses were conducted to explore the impact of key assumptions on the cost-effectiveness of case finding and were pre-specified by the team. These were as follows: including a utility decrement for women whose case-finding outcome was a false positive (2% and 10%), alternative durations of midwife time required to administer and score case-finding instruments (0 min if done online prior visit; three times the duration observed in the BaBY PaNDA study to allow a more conversational approach: 5.13 min for the Whooley questions and 10.62 min for the PHQ-9 or EPDS), and alternative resource use associated with false positive cases initiating treatment (10% and 30% of the full treatment/monitoring cost for mild depression).

In previous cost-effectiveness models, it was assumed that there was an 8.3% chance that women who had a false negative case-finding outcome would be subsequently identified as depressed by their GP (National Institute of Health and Care Excellence (NICE) 2014, p. 192; Littlewood et al. 2018). This figure was derived from observational data from a single GP surgery, collected in 1997 (Kessler et al. 2002). We felt that this was unlikely to reflect current practice and so sought expert opinion. Based on this, a value of 10% was used in our base case model and sensitivity analyses explored alternative values of 5% and 25%. Also, in our base case model, we have assumed that in the absence of a case-finding programme, GP appointments to assess pregnant women presenting with symptoms of depression would be of average length (9.22 min (Jones, KC and Burns, A 2021)). However, experts (clinical and by lived experience) suggested that this is likely to be an underestimation. Typically, this appointment would be 10–15 min long if by telephone or 15–20 min face-to-face. Sensitivity analyses explored these alternative parameters.

### Stakeholder involvement and engagement

We brought together a group of mothers (*n* = 3) who had lived experience of perinatal mental illness. They were introduced to basic concepts of cost-effectiveness analysis in relation to healthcare and how cost-effectiveness evidence can inform decision making. The group was provided with an overview of planned methods including the model structure and asked for their input. Prior to this exercise, we were not planning on including the PHQ-9 as a one-stage strategy. However, the experience of the group suggested that this was sometimes used in practice already and so we explored it as part of our analysis. It was not included in the final analysis because its sensitivity was less than 70%. Consulting with experts has enabled us to better reflect current practice in the NHS in the parameters of our model.

## Results

There were 391 participants in the BaBY PaNDA study cohort who had provided data on at least one of the antenatal depression risk factors (summarised in Table 2). Over half of this group (55.8%) had one or more risk factor for antenatal depression at baseline assessment. The most common risk factors in the sample were a history of either anxiety and/or depression.

**Table 2:** Summary of antenatal depression risk factors in the BaBY PaNDA study cohort

Sample characteristics	Women with data on risk factors*n* = 391
Age at consent (years), *mean (SD)*	31.2 (5.1)
Aged < 20 years at consent, *n (%)*	11 (2.8)
History of anxiety, *n (%)*	138 (35.3)
History of depression, *n (%)*	133 (34.0)
One or more threatening life event*, *n (%)*	71 (18.2)
One or more antenatal depression risk factor, *n(%)*	218 (55.8)

*According to response on List of Threatening Events Questionnaire (LTE-Q); LTE-Q data available for 245 participants

Table 3 shows the costs and QALYs associated with the three different case-finding strategies in the high-risk group (top half of the table) and for the whole sample (bottom half of the table). When the analysis was restricted to women at high risk of developing antenatal depression, the two-stage strategy of Whooley questions followed by PHQ-9 had the lowest cost. It was also associated with the smallest number of QALYs compared with the other 2 strategies evaluated. However, the additional cost associated with the EPDS-10 or Whooley questions as one-stage strategies was above the cost-effectiveness threshold of £20,000, and so, these strategies are unlikely to be cost-effective compared with the two-stage (Whooley/PHQ-9) strategy.

**Table 3:** Costs and QALYs of case-finding strategies in women at high risk of antenatal depression and whole sample and cost-effectiveness of the most cost-effective strategy versus no case finding

Strategy		Mean cost per person (£)	Mean QALYs per person		ICER (£)*		
High risk subgroup							
Whooley questions followed by PHQ-9		82.37	0.3275		-		
EPDS-10		89.90	0.3277		42,053		
Whooley questions		92.76	0.3277		Dominated		
Whole sample							
Whooley questions followed by PHQ-9		52.06	0.3459		-		
EPDS-10		58.64	0.3461		33,246		
Whooley questions		60.92	0.3461		Dominated		
Strategy	Mean cost per person (£) (95% CI)	Mean QALYs per person (95% CI)	ICER* (£)	Probability of cost-effectiveness for maximum WTP			
				£0		£20,000	£30,000
Whooley → PHQ-9	82.37 (61.71, 105.96)	0.3275 (0.3160, 0.3392)	3538	0.398		0.783	0.805
No case finding	80.17 (67.47, 94.75)	0.3269 (0.3152, 0.3387)		0.602		0.217	0.195

When there is no case finding, women can only be identified as having PND if they visit their GP

*QALYs* quality adjusted life years, *ICER* incremental cost-effectiveness ratio, *WTP* willingness to pay

*Mean costs/QALYs reported are rounded values whereas ICERs are calculated based on unrounded values

Sensitivity analyses are reported in full in supplementary material (Table S2). When a health utility decrement was included for people who received a false positive case-finding outcome, the health benefit for the two-stage strategy became larger than the other 2 strategies and so it dominated both of them as it was the least costly. When more time to administer the case-finding instruments was included (three times longer than in the base case model to allow for a more conversational approach), the cost of the Whooley questions strategy increases by less than the other 2 strategies. As a result, the cost required to achieve the additional health gained compared to the two-stage (Whooley/PHQ-9) strategy now falls below the £20,000 cost-effectiveness threshold (£15,516/QALY) and so would be considered cost-effective. The other sensitivity analyses did not affect the results.

When the whole sample was included in the analysis, costs were lower and QALYs were higher, but the overall results were the same as for the high-risk group (i.e. the two-stage strategy was the most cost-effective option).

Table 3 also shows the costs and QALYs for the Whooley/PHQ-9 strategy compared with no case finding in the high-risk group only. There is a small additional cost and QALY gain associated with case finding, resulting in an ICER of £3538. The ICER is well below a threshold of £20,000/QALY and there is a high (78%) chance that at this threshold case finding would be cost-effective compared to no case finding. If decision-makers had a willingness to pay threshold of £0, the likelihood that case finding would be the preferred option falls to 40%.

The estimated outcomes of implementing the case-finding strategies for a 1-year cohort of women having a baby in England are presented in Table 4. The breakdown of outcomes (true positive, true negative, false negative, and false positive) demonstrates where the costs and health benefits arise in the model. For example, the number of true positives is highest for the targeted case-finding strategy, which is a positive thing as the aim is to improve identification of true positives. However, the number of false positives is highest for this strategy, so more people are initiating treatment that they do not need and will not benefit from (i.e. money is spent on treatment with no increase in health to offset this cost). The number of false positives is by far the lowest for universal case finding; when compared with no case finding, there are 16,154 fewer false positives (pregnant women diagnosed with depression when they visit a GP under this option). What is also notable in Table 4 is the low prevalence of antenatal depression in the low-risk group (1.7%) compared with the high-risk group (17.1%) which suggests that the risk factors used to classify the sample are appropriately identifying those are high/low risk.

**Table 4:** Sensitivity, specificity, resources, and outcomes in a hypothetical cohort of 600,000 women

Strategy	No case finding	Whooley → PHQ-9 universal	Whooley → PHQ-9 targeted	
			High risk	Low risk
Prevalence of depression	10.3%	10.3%	17.1%	1.7%
Sensitivity	50.1%	70.6%	74.2%	50.1%
Specificity	81.3%	89.5%	85.0%	81.3%
Proportion who visit GP with antenatal depression symptoms	72.2%	0%	0%	64.2%
Number of people	600,000	600,000	334,800	265,200
Number with depression	61,800	61,800	57,251	4549
True positives	30,962	43,631	42,480	2279
True negatives	465,535	481,689	235,917	229,359
False negatives	30,838	18,169	14,771	2270
False positives	72,665	56,511	41,632	31,292
Total costs	36,616,885	31,240,707	37,136,402	
Total QALYs	207,342	207,546	207,567	
Mean costs and QALYs (per person)*				
Costs	61.10 (49.51–75.60)	52.24 (33.65–74.13)	61.81 (49.45–76.51)	
QALYs	0.3455 (0.3331–0.3580)	0.3458 (0.3331–0.3587)	0.3459 (0.3336–0.3583)	
ICER (£/QALY)**	Dominated by universal	-	163,763 (vs. universal)	
Probability cost-effective at maximum WTP*:				
£0/QALY	0.042	0.747	0.210	
£20,000/QALY	0.049	0.557	0.394	
£30,000/QALY	0.066	0.515	0.419	

*QALYs* quality adjusted life years, *ICER* incremental cost-effectiveness ratio, *WTP* willingness to pay

*Based on 10,000 iterations

**Costs and QALYs reported are rounded values, whereas ICERs are calculated based on non-rounded values

**Fig. 2:**
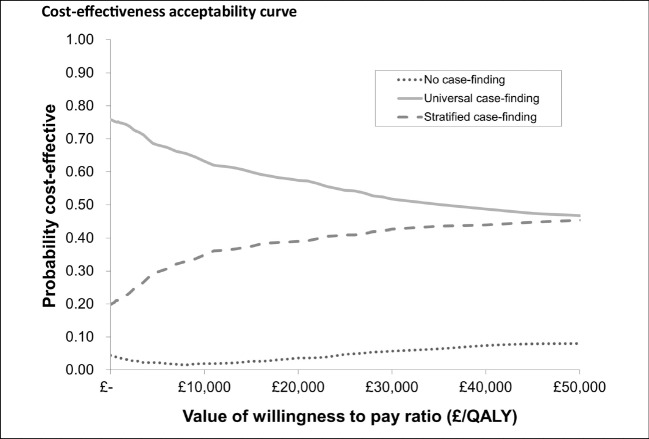
Cost-effectiveness acceptability curve

The mean cost per person for universal case finding is lowest of the three options (£52), followed by no case finding (£61) and then targeted case finding (£62), although it should be noted that the cost of targeted and no case finding are very close. Both case-finding strategies improve health compared with no case finding and so because universal case finding is cost-saving this dominates no case finding (i.e. less money for more health benefit). At a cost of £163,763/QALY, the small additional health benefit associated with targeted case finding compared with universal case finding is not good value for the additional spend required. At willingness to pay threshold between £0 and £30,000, there is a very low probability (4–7%) that no case finding is likely to be cost-effective compared to either of the case-finding programmes. This is summarised graphically in Fig. 2 which shows cost-effectiveness acceptability curves for the three strategies across a range of willingness to pay thresholds.

The results of key sensitivity analyses are presented in Table 5 (all sensitivity analyses are reported in Supplementary Material; Table S3). Incorporating a utility decrement for women with a false positive case-finding outcome strengthens the case for the universal programme as it then also dominates the targeted approach (due to higher QALY gains). When more time is allowed to conduct case finding, the universal programme no longer dominates no case finding because of the higher costs; however, the size of the health gain associated with case finding means that universal case finding would still be considered cost-effective compared with no case finding (£2296/QALY). Although it does not change the results (i.e. universal case finding still dominates), when longer GP consultations to assess depression are included in the model, no case finding becomes the most expensive option.

**Table 5:** Results of key sensitivity analyses for a universal case-finding strategy using two-stage PHQ-9 (based on 10,000 iterations)

	Mean cost per person (£)	Mean QALYs per person	ICER (£/QALY)	Probability of cost-effectiveness at £20,000/QALY
*Base case analysis*: 10% likelihood of being identified in primary care following false negative outcome; no utility decrement for false positive cases				
Universal	52.24	0.3458	-	0.557
No case finding	61.10	0.3455	Dominated	0.049
Targeted	61.81	0.3459	163,763	0.394
Utility decrement associated with a false positive outcome				
Utility decrement—2%				
Universal	52.24	0.3451	-	0.049
No case finding	61.10	0.3450	Dominated	0.588
Targeted	61.81	0.3446	Dominated	0.363
Utility decrement—10%				
Universal	52.24	0.3425	-	0.656
No case finding	61.10	0.3412	Dominated	0.094
Targeted	61.81	0.3416	Dominated	0.250
Time to conduct case finding				
Allow more time to administer and score case-finding instruments (3 × midwife time)				
No case finding	61.10	0.3455	-	0.151
Universal	61.90	0.3458	2296	0.431
Targeted	67.20	0.3459	90,692	0.417
Longer GP consultation to assess depression with no screening				
1.5 times the 9.22 min average appointment time (13.83 min)				
Universal	52.63	0.3458	-	0.588
Targeted	62.32	0.3459	165,811	0.403
No case finding	76.50	0.3455	Dominated	0.009

*GP* general practitioner, *QALYs* quality-adjusted life years, *ICER* incremental cost-effectiveness ratio

## Discussion

In our secondary analysis of data from an observational cohort study of pregnant women, we found that the prevalence of antenatal depression was 10 times higher in women identified as being at high-risk for depression (vs. rest of sample). When only the high-risk subgroup are considered, case finding using a two-stage Whooley/PHQ-9 strategy is cost-effective compared with no case finding. The additional cost to improve health by one QALY was £3538 and the likelihood of case finding being cost-effective compared with no case finding was 78% (if decision-makers are willing to pay £20,000/QALY). However, universal case finding for antenatal depression (i.e. case finding with all pregnant women) appears to be an even better option. Compared with no case finding, universal case finding costs less and improves the health of pregnant women more (i.e. universal case finding dominates no case finding). The only scenario explored where no case finding was not dominated by universal case finding was when the amount of time included to conduct case finding was increased threefold. Even in this scenario, the additional cost associated with universal case finding to improve health by one QALY was £2296 which is well below typically cited thresholds (NICE 2022).

With targeted case finding, the proportion of true positive outcomes (i.e. depression correctly identified) is highest (vs. universal case finding or no case finding), and false negative outcomes (i.e. missed cases) is lowest. However, targeted is less cost-effective than universal case finding because there are considerably more false positive outcomes with a targeted approach who are subsequently treated, and treatment is a key driver of cost in the model. Although, varying the cost of treating false positive cases had minimal impact on the ICER and there were no scenarios explored where targeted case finding was more cost-effective than universal case finding. However, the choice of screening strategy also needs to account for feasibility and implementation concerns (e.g. healthcare practitioner burden) as well as other types of evidence around pregnant women’s preferences for case finding (e.g. qualitative evidence).

Another benefit of universal case finding is that it does not require any additional ‘targeting’ process to identify women at high risk of antenatal depression. Three of the factors (age, history of depression, history of anxiety) are routinely collected as part of standard antenatal care. This means that they are readily available in medical notes/records and can be identified easily and with no additional resources required. Capturing the fourth risk factor, whether someone has experienced a recent adverse life event, may be more complex to operationalise in practice. Identifying aspects of patient heterogeneity that can be targeted and implemented in practice is a noted issue in cost-effectiveness analysis (Shields et al. 2022).

The BaBY PaNDA study also found that antenatal depression case finding with a two-stage Whooley/PHQ-9 strategy was associated with lower costs and higher QALYs than no case finding and that at a threshold of £20,000/QALY, this strategy was most likely to be cost-effective (Littlewood et al. 2018). Our findings are also supported by those from another decision-tree model analysis which found that case finding (or screening as termed in that study) was more likely to be cost-effective than no case finding at a willingness to pay threshold of £20,000/QALY (Heslin et al. 2022). A key strength of that model is that the parameters regarding the sensitivity and specificity of case-finding instruments were taken from a large participant sample, although all participants lived in London. The same ‘gold standard’ comparator for the performance of case-finding instruments (the SCID) was used for the BaBY PaNDA data. The utility values used in that model were also taken from the BaBY PaNDA study (albeit without stratification by depression risk factors like in our study) as were the costs of conducting case finding. Other model parameters were also taken from the same source as our study including the probability of treatment response. The other model only included 2 types of treatment in their model, different intensities of psychological therapy, whereas we included a pharmacological treatment (as there are SSRIs which are safe for use in pregnancy) and we felt that it was important to consider the treatment options that would be potentially offered to pregnant women in a real-world setting. Key differences were that that study did not evaluate the PHQ-9 as part of either a one- or two-stage strategy, did not consider a targeted case-finding strategy, and focussed on a slightly different time period (i.e. from 8–10 weeks gestation to 3 months postpartum compared with 20–40 weeks gestation in the current study). The findings from a previous model comparing the cost-effectiveness of universal and targeted case finding for *postnatal* depression also suggested that a universal strategy was more likely to be cost-effective than a targeted strategy (Camacho et al. 2023).

### Strengths and limitations

Improving the identification of antenatal depression is important because, for some women, untreated depression can lead to poor outcomes for mother and baby (Slomian et al. 2019). A strength of our model is that we included different utility values for women with mild versus moderate-to-severe depression. This meant that we were able to incorporate the assumption that for women whose antenatal depression remained undetected (and who did not spontaneously recover), their utility values were initially equivalent to mild depression, but then these worsened over time to moderate-to-severe depression. Another related strength is that we excluded case-finding strategies which did not reach an acceptable threshold for sensitivity (i.e. correctly identifying cases of antenatal depression). The cost-effectiveness of strategies which are not good at identifying true positive cases is driven by keeping treatment costs low at the population level. This is in conflict with the aim of the case-finding programme (i.e. to improve the identification of antenatal depression and enable more women to access treatment).

Key limitations of our model include the time horizon and the perspective. The time horizon covers the period from the time of screening (approximately 20 weeks gestation) to around the time of birth and so does not account for the costs or health impacts of persistent depression into and beyond the postnatal period. The perspective of the model includes only the health impacts for mothers and does not include impacts on other family members (e.g. impact on partners or birth outcomes). Similarly, the model does not incorporate the impact of antenatal experiences of depression on the likelihood of women developing depression (or other mental health conditions) in the postnatal period. The longer-term and broader impacts of mental health problems in the perinatal period are estimated to be considerable (Bauer et al. 2016). Another limitation of the model is that it assumes that case finding takes place at around 20-weeks’ gestation. This is when data on the case-finding instruments and clinical diagnosis were collected in the BaBY PaNDA study. The model does not explore cost-effectiveness of case finding at more than one time point, or whether earlier or later in pregnancy would have an impact on cost-effectiveness.

The treatment pathways included in the model were based on national guidance in England (National Institute of Health and Care Excellence (NICE) 2014), and data reported in a publication from the English-based Born in Bradford (BiB) cohort study (Prady et al. 2016). The BiB study reported the proportion of people receiving different treatments for common mental disorder during pregnancy. Strengths of the BiB data were that they were recent and based on a large number of real-world observations and so likely to reflect current real-world practice. A limitation of the data from the BiB study is that they included a broader group of mental health conditions than depression alone, and so, they may not be fully representative. A limitation of our model is that the probability of treatment efficacy was derived from studies of women with postnatal rather than antenatal depression. It is possible that efficacy is different at these time points, but there have been far fewer studies in pregnant women on which to base model parameters, and so, it was necessary to make this assumption. While our model included three treatment options (pharmacological, non-pharmacological, or a combination of both), it did not allow for people to try more than one option in sequence and did not account for engagement with treatment and the impact this may have on likelihood of treatment response. Robust primary data on treatment pathways and efficacy in this population may allow the use of cost-effectiveness models which incorporate more individual variation in treatment pathways (e.g. discrete event simulation).

Data from the BaBY PaNDA study were used to estimate a number of parameters in the model including the prevalence of antenatal depression and of the depression risk factors. The BaBY PaNDA sample were recruited predominantly from Yorkshire in the North of England and included areas of varying sociodemographic characteristics (e.g. economic deprivation), although they were predominantly (98%) of white ethnicity. The prevalence of antenatal depression in the whole BaBY PaNDA sample was 10.3% and notably higher in the high-risk subgroup (17.1%). An umbrella review reported that the global prevalence of antenatal depression was between 15 and 65%; however, this included estimates from studies that did not use standard quality appraisal criteria (Dadi et al. 2020). A systematic review of high-quality studies reported the prevalence of antenatal depression within the range of 4.8% and 33.2%, with a mean of 17.2% (Underwood et al. 2016). Another systematic review meta-analysed the prevalence of any antenatal depression and major antenatal depression (Yin et al. 2021). They reported a pooled prevalence of any antenatal depression of 20.7% (95% CI 19.4–21.9%) and major antenatal depression 15.0% (95% CI 13.6–16.3%). The prevalence of antenatal depression in the BaBY PaNDA sample was in the lower range reported in the published reviews. However, when this was restricted to studies which used a clinical interview to identify cases of depression, the pooled estimate reported by Yin et al. was 12.6% (95% CI 10.3–14.9) which is much closer to the estimate from BaBY PaNDA (which was based on clinical interviews) (Yin et al. 2021). A larger UK-based study which was not only included in that meta-analysis but also used clinical interviews to identify antenatal depression reported a prevalence of 11%, again very similar to the estimate from BaBY PaNDA (Howard et al. 2018). More than half of the BaBY PaNDA sample were categorised as being at high-risk of antenatal depression. However, we were unable to find data from other studies to indicate whether this was representative of the general population. The parameters derived from BaBY PaNDA data may not be fully generalisable to the rest of England, particularly inner-city areas in the South. Having said this, the scenario with the highest ICER for universal case finding is so far below the £20,000/QALY cost-effectiveness threshold that even if the margin of difference was three- or fourfold and the ICERs trebled or quadrupled, they would still be below the £20,000 threshold.

*Ethical/equity considerations.*

Universal case finding for antenatal depression may address some barriers related to stigma around mental illness, as it may be more acceptable that all pregnant women are asked to respond to the same questions routinely and as part of standard care. Case finding for antenatal depression is also potentially less subject to bias than current guidance in England which advise that healthcare providers subjectively consider asking pregnant women about their mental health. However, case-finding instruments may have different sensitivity and specificity across sociocultural groups. An important consideration is whether there is a need to develop culturally adapted case-finding instruments, and it is vital that this work is done in partnership with members of the respective communities. It may also be the case that the factors we used to categorise pregnant women as high-risk may differentially predict antenatal depression in minority ethnic populations. Future work to better understand the potentially complex relationship between minoritised ethnicity and risk of antenatal depression is needed. Future observational studies should aim to recruit representative samples. The likelihood of response to treatment for depression will be related to acceptability and adherence to treatment. It is possible that socio-cultural factors may influence this, for example, limited English and mental health literacy may be a barrier to people in following non-adapted facilitated self-help programmes. Distributional cost-effectiveness analysis (DCEA) in future economic evaluation may help to provide information on the equity of cost and benefit distribution (Cookson et al. 2017).

## Conclusion

Case finding is a cost-effective way of improving the detection of antenatal depression and improving the health of pregnant women from the perspective of the English NHS and social care services. If integrated as part of routine antenatal care, it could improve the identification of antenatal depression, reduce health inequalities (through standardisation of care) and potentially reduce costs within the health service. However, it is recognised that both staffing and financial resources within a health service are finite and are important considerations for the successful implementation of a case-finding programme.

## Supplementary information

Below is the link to the electronic supplementary material.

Supplementary file1 (DOCX 208 KB)

## Data Availability

Model parameters are presented in full in Supplementary Material. Data from the BaBY PaNDA study which was used to derive some model parameters (as a secondary analysis) are available upon reasonable request from the BaBY PaNDA study team (co-author Gilbody led the BaBY PaNDA study).
