# Supplementary material for: An economic evaluation of universal and targeted case-finding strategies for identifying antenatal depression: a model-based analysis comparing common case-finding instruments
Source: Arch Womens Ment Health. 2023 Oct 18;28(4):853–67. doi: 10.1007/s00737-023-01377-2 (PMC12283817; doi:10.1007/s00737-023-01377-2)
Supplement: Supplementary file 1 — Supplementary file1 (DOCX 208 KB) [file 737_2023_1377_MOESM1_ESM.docx]

**An economic evaluation of universal and targeted case-finding strategies for identifying antenatal depression: a model-based analysis comparing common case-finding instruments**

**Supplementary material**

**Figure S1.** Decision tree structure for risk stratification

**
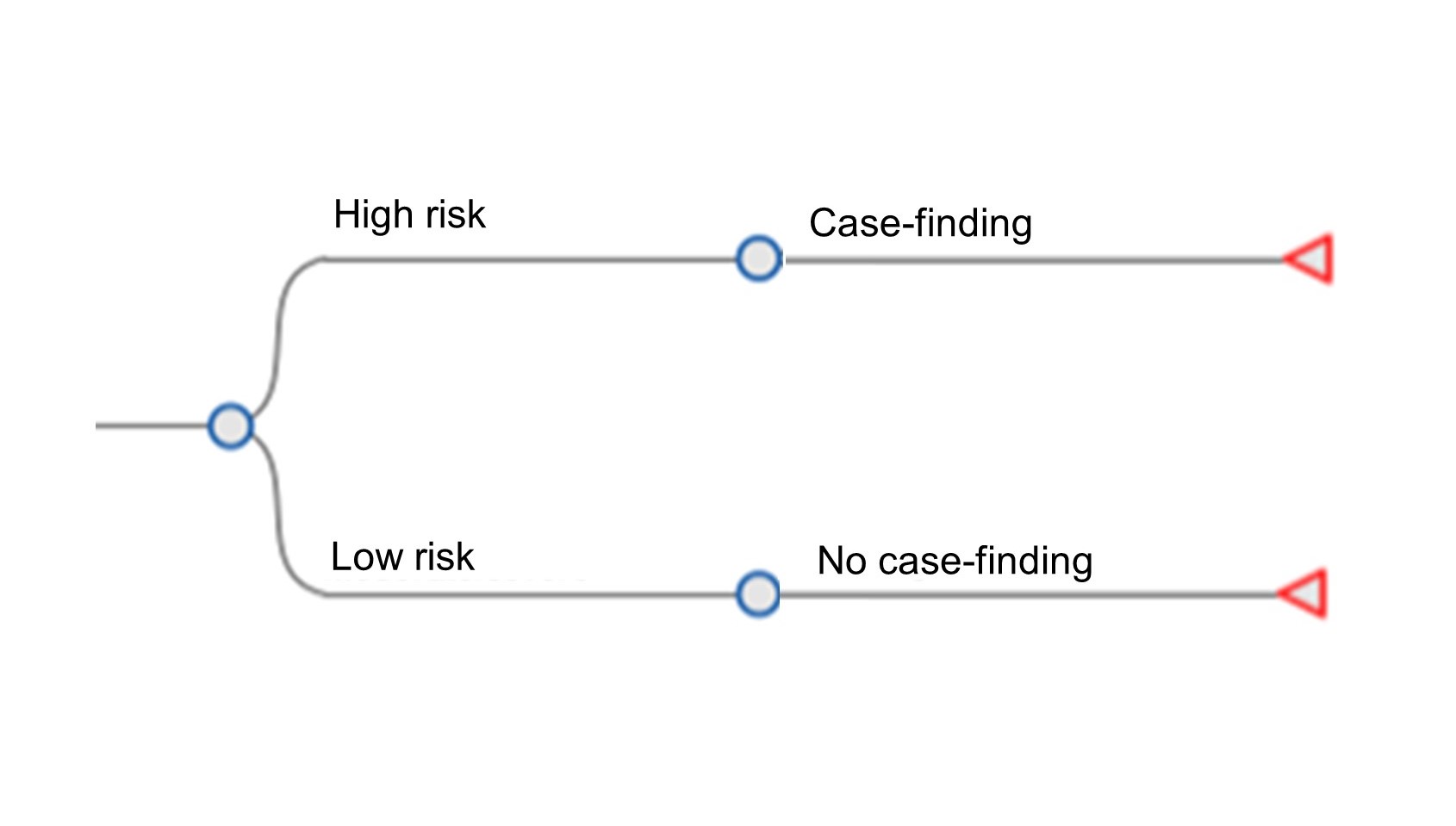
**

**Figure S2.** Decision tree structure for case-finding and treatment


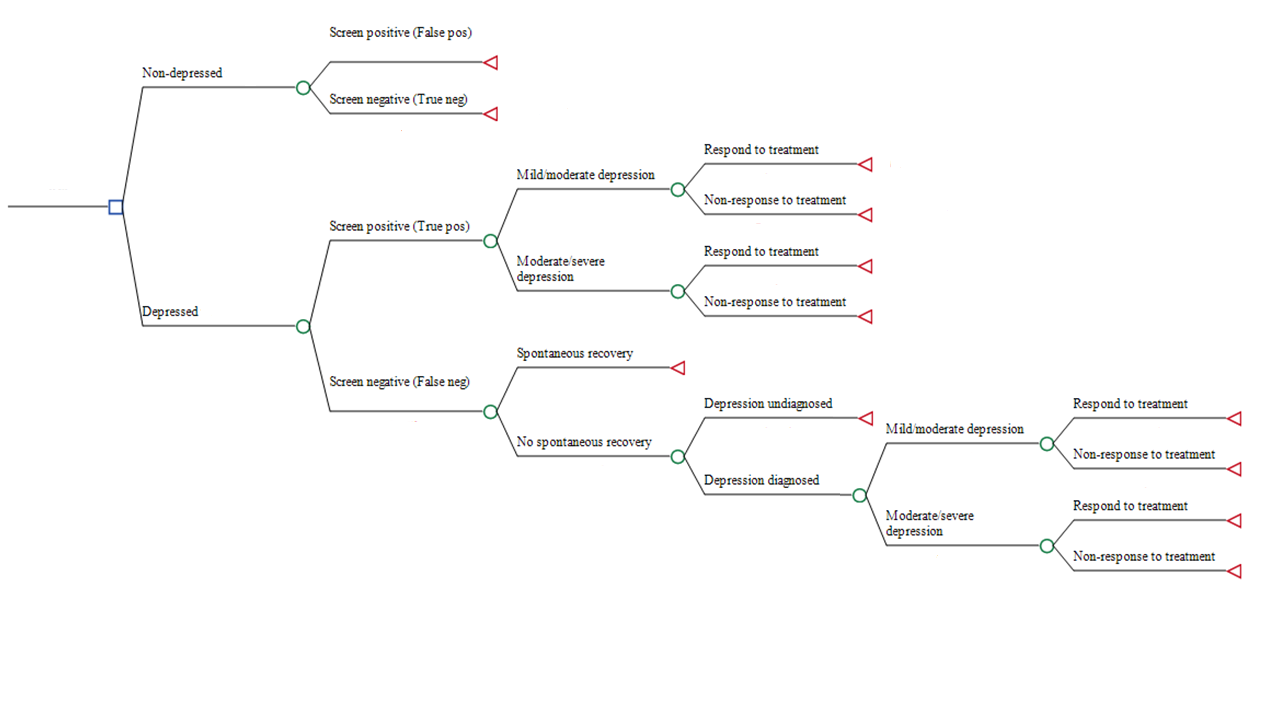


*Sensitivity and specificity of case-finding* *strategies in high-risk women*

The top section of Table S1 shows the sensitivity and specificity of the potential case-finding strategies in high-risk women from the BaBY PaNDA sample. A sensitivity greater than 70% and a specificity greater than 60% were considered acceptable. As such, the cost-effectiveness of the EPDS-10, Whooley questions, and Whooley questions followed by PHQ-9 were estimated. The EPDS-13, PHQ-9, and Whooley questions followed by EPDS-13 were excluded as their sensitivity was less than 70%. The Whooley questions followed by EPDS-10 was excluded because its specificity was less than 60%. The lower section of Table S1 shows the sensitivity and specificity of the potential case-finding strategies across the whole BaBY PaNDA sample; the acceptable strategies are the same as for the high-risk subgroup.

**Table S1. Sensitivity and specificity of case-finding strategies in high-risk sub-group and the whole sample**

| Strategy | Sensitivity (95% CI) | Specificity (95% CI) | Acceptable (sensitivity>70% AND specificity >60%) |
| --- | --- | --- | --- |
| High-risk sub-group | | | |
| EPDS-10 | 81.1%  (64.8% to 92.0%) | 80.6%  (74.0% to 86.1%) | Yes |
| EPDS-13 | 45.9%  (29.5% to 63.1%) | 93.3%  (88.6% to 96.5%) | No |
| Whooley | 83.8%  (68.0% to 93.8%) | 77.8%  (71.0% to 83.6%) | Yes |
| PHQ-9 | 67.6%  (50.2% to 82.0%) | 95.0%  (90.7% to 97.7%) | No |
| EPDS-10 (after Whooley) | 90.3%  (74.2% to 98.0%) | 37.5%  (22.7% to 54.2%) | No |
| EPDS-13 (after Whooley) | 51.6%  (33.1% to 69.8%) | 72.5%  (56.1% to 85.4%) | No |
| PHQ-9 (after Whooley) | 74.2%  (55.4% to 88.1%) | 85.0%  (70.2% to 94.3%) | Yes |
| Whole sample | | | |
| EPDS-10 | 82.5%  (67.2% to 92.7%) | 86.6%  (82.5% to 90.0%) | Yes |
| EPDS-13 | 45.0%  (29.3% to 61.5%) | 95.7%  (93.0% to 97.6%) | No |
| Whooley | 85%  (70.2% to 94.3%) | 83.7%  (79.4% to 87.4%) | Yes |
| PHQ-9 | 65%  (48.3% to 79.4%) | 97.1%  (94.8% to 98.6%) | No |
| EPDS-10 (after Whooley) | 91.2%  (76.3% to 98.1%) | 43.9%  (30.7% to 57.6%) | No |
| EPDS-13 (after Whooley) | 50%  (32.4% to 67.6%) | 77.2%  (64.2% to 87.3%) | No |
| PHQ-9 (after Whooley) | 70.6%  (52.5% to 84.9%) | 89.5%  (78.5% to 96%) | Yes |

**Table S2. Results of sensitivity analyses in the sub-group of women at high risk of antenatal depression**

| **Strategy** | **Mean cost per person (£)** | **Mean QALYs per person** | **ICER (£)*** |
| --- | --- | --- | --- |
|  |  |  |  |
| Base case analysis: recovery to utility value of non-depressed women in high-risk group; no utility decrement for false positive cases; in-person administration and scoring of case-finding instruments; cost of initiating treatment in false positive cases is 20% of the full treatment cost | | | |
| Whooley questions followed by PHQ-9 | 82.37 | 0.3275 | - |
| EPDS-10 | 89.90 | 0.3277 | 42,053 |
| Whooley questions | 92.76 | 0.3277 | Dominated |
| Sensitivity analyses | | | |
| Additional utility decrement following false positive outcome - 2% | | | |
| Whooley questions followed by PHQ-9 | 82.37 | 0.3267 | - |
| EPDS-10 | 89.90 | 0.3266 | Dominated |
| Whooley questions | 92.76 | 0.3265 | Dominated |
| Additional utility decrement following false positive outcome - 10% | | | |
| Whooley questions followed by PHQ-9 | 82.37 | 0.3233 | - |
| EPDS-10 | 89.90 | 0.3223 | Dominated |
| Whooley questions | 92.76 | 0.3216 | Dominated |
| Online administration and scoring of case-finding instruments (zero midwife time to administer) | | | |
| Whooley questions followed by PHQ-9 | 77.54 | 0.3275 | - |
| EPDS-10 | 86.64 | 0.3277 | 50,820 |
| Whooley questions | 91.19 | 0.3277 | Dominated |
| Allow more time to administer and score case-finding instruments (3x midwife time) | | | |
| Whooley questions followed by PHQ-9 | 92.03 | 0.3275 | - |
| Whooley questions | 95.90 | 0.3277 | 15,516 |
| EPDS-10 | 96.42 | 0.3277 | Dominated |
| Resource use in women with a false positive case-finding outcome – 10% of true positive | | | |
| Whooley questions followed by PHQ-9 | 78.95 | 0.3275 | - |
| EPDS-10 | 85.48 | 0.3277 | 36,452 |
| Whooley questions | 87.70 | 0.3277 | Dominated |
| Resource use in women with a false positive case-finding outcome – 30% of true positive | | | |
| Whooley questions followed by PHQ-9 | 85.79 | 0.3275 | - |
| EPDS-10 | 94.32 | 0.3277 | 47,654 |
| Whooley questions | 97.82 | 0.3277 | Dominated |
| *Mean costs and QALYs reported are rounded values whereas ICERs are calculated based on unrounded values  QALYs = quality adjusted life years; ICER = incremental cost-effectiveness ratio | | | |

**Table S3. Results of sensitivity analyses in all antenatal women**

|  | **Mean cost per person (£)** | **Mean QALYs per person** | **ICER (£/QALY)** | **Probability of cost-effectiveness at £20,000/QALY** |
| --- | --- | --- | --- | --- |
| Base case analysis: 10% likelihood of being identified in primary care following false negative outcome; no utility decrement for false positive cases | | | | |
| Universal | 52.24 | 0.3458 | - | 0.557 |
| No case-finding | 61.10 | 0.3455 | Dominated | 0.049 |
| Targeted | 61.81 | 0.3459 | 163,763 | 0.394 |
| **Likelihood of being identified in primary care following false negative case-finding outcome** | | | | |
| Proportion of women with false negative result later identified in routine care – 5% | | | | |
| Universal | 51.82 | 0.3458 | - | 0.556 |
| No case-finding | 60.38 | 0.3454 | Dominated | 0.049 |
| Targeted | 61.42 | 0.3458 | 159,255 | 0.394 |
| Proportion of women with false negative result later identified in routine care – 25% | | | | |
| Universal | 53.49 | 0.3459 | - | 0.558 |
| No case-finding | 63.26 | 0.3456 | Dominated | 0.050 |
| Targeted | 63.00 | 0.3459 | 188,344 | 0.392 |
| **Utility decrement associated with a false positive outcome** | | | | |
| Utility decrement - 2% | | | | |
| Universal | 52.24 | 0.3451 | - | 0.049 |
| No case-finding | 61.10 | 0.3450 | Dominated | 0.588 |
| Targeted | 61.81 | 0.3446 | Dominated | 0.363 |
| Utility decrement - 10% | | | | |
| Universal | 52.24 | 0.3425 | - | 0.656 |
| No case-finding | 61.10 | 0.3412 | Dominated | 0.094 |
| Targeted | 61.81 | 0.3416 | Dominated | 0.250 |
| **Resource use associated with a false positive outcome** | | | | |
| Proportion of true positive cost - 10% | | | | |
| Universal | 49.65 | 0.3458 | - | 0.560 |
| No case-finding | 57.78 | 0.3455 | Dominated | 0.054 |
| Targeted | 59.89 | 0.3459 | 175,213 | 0.386 |
| Proportion of true positive cost - 30% | | | | |
| Universal | 54.83 | 0.3458 | - | 0.553 |
| No case-finding | 64.43 | 0.3455 | Dominated | 0.046 |
| Targeted | 63.74 | 0.3459 | 152,312 | 0.401 |
| **Time to conduct case-finding** | | | | |
| EPDS completed online – no midwife time required | | | | |
| Universal | 47.41 | 0.3458 | - | 0.592 |
| Targeted | 59.12 | 0.3459 | 200,298 | 0.379 |
| No case-finding | 61.10 | 0.3455 | Dominated | 0.029 |
| Allow more time to administer and score case-finding instruments (3x midwife time) | | | | |
| No case-finding | 61.10 | 0.3455 | - | 0.151 |
| Universal | 61.90 | 0.3458 | 2,296 | 0.431 |
| Targeted | 67.20 | 0.3459 | 90,692 | 0.417 |
| **Longer GP consultation to assess depression with no screening** | | | | |
| 1.5 times the 9.22 minute average appointment time (13.83 minutes) | | | | |
| Universal | 52.63 | 0.3458 | - | 0.588 |
| Targeted | 62.32 | 0.3459 | 165,811 | 0.403 |
| No case-finding | 76.50 | 0.3455 | Dominated | 0.009 |
| 2 times the 9.22 minute average appointment time (18.44 minutes) | | | | |
| Universal | 53.02 | 0.3458 | - | 0.594 |
| Targeted | 62.83 | 0.3459 | 167,860 | 0.404 |
| No case-finding | 91.88 | 0.3455 | Dominated | 0.002 |
| QALYs = quality adjusted life years; ICER = incremental cost-effectiveness ratio | | | | |
